# On Reading Medical Books

**Published:** 1804-12-01

**Authors:** 


### *To the Editors of the Medical and Physical Journal.*

Gentlemen,

As any thing which relates to the improvement of medical science is readily admitted into your publication, possibly you will insert this from a constant Reader of your communicative and useful works.

Being one of the medical profession, and constantly anxious to cultivate that science in the most proper manner, ner, much of my time has been occupied in ascertaining this point.

I am at present a young man, lately settled in practice, after having studied with the greatest assiduity Physic, Surgery, and their necessary branches, in London and at other places, for a considerable number of years, and have now in particular a great propensity to *read* every publication relating to my profession, from which intelligence may be gained. I am convinced of the truth of the old observation, "Est modus in rebus,' and wish to act as influenced by it. I have frequently endeavoured to prove the propriety of reading medical works, in conversation with different men of my profession, and as frequently almost found them ready to oppose me; suffer me, therefore to make a few observations relative to *reading in particular*, as one mode of instruction.

The general objection started against reading, is, that it is likely to make a man an unsteady practitioner or a theorist. This savors too much of the natural disposition of the English, who had rather follow the tracks of their ancestors, than endeavour, by theory and experiment, to improve a science which, from a due observation of facts, might daily be brought nearer to perfection; as a wise author justly observes,—"Prejudice often prevents men from seeing the truth, though it stands before them."

*Reading* without theorizing, I consider as but of little use; and I am sometimes inclined to think, that those who declaim against it, are too idle to think or to arrange important matter which might be acquired from reading, so as to retain it, and apply it when necessary; thereby verifying the old proverb, "Quod volumus facile credimus."

An elderly man perhaps will say, When I was young, I read much, paid great attention to what I read, and if I met with any new medicine, or any new mode of treating a disease, recommended, I gave it a trial; but finding them in time give place to other new doctrines, I went back to my old plan, and so have proceeded for years. This was language really made use of to me by a respectable practitioner, considered eminent by the people among whom he resided. I am vexed to meet with old professors conversing in this way to students, because I am convinced it will lead many young minds astray, if too much impressed with the opinion of men who have become idle with old age.

Mr. John Hunter is generally brought forward by some, as an instance of an eminent man who read but little; but but it should be at the same time remembered, that he was a man of strong natural abilities, such as few possess, and of intense thought.

I would only ask, how it is possible the practice of a medical man should keep pace with the improvements of his profession, where he resided in the country, without reading? One of the most useful kinds of reading, I consider that of reading periodical publications like the Medical and Physical Journal, for the papers in this work, be they ever so disinteresting, are not of sufficient length to occupy much of the reader's time; and, in general, are so well selected as to convey much intelligence, and to lead the reader into a train of ideas upon a variety of subjects, from which great benefit might arise, and which otherwise would have been neglected. I am convinced much more might be said in favour of *reading*; but I do not wish to occupy much of your paper, trusting that some one of your Correspondents, more competent than myself, will favour the public with some further remarks on this subject, or point out his objections to these of

*September* 6, 1804. JUVENIS.

